# *“I try the one that they say is good.”* - factors influencing choice of health care provider and pathways to diabetes care for Syrian refugees in Lebanon

**DOI:** 10.1186/s13031-021-00375-4

**Published:** 2021-06-05

**Authors:** Flora Haderer, Emilie Venables, Josefien van Olmen, Miriam Orcutt, Michella Ghassibe-Sabbagh, Wilma van den Boogaard

**Affiliations:** 1 Médecins Sans Frontières Operational Centre Brussels, Brussels, Belgium; 2 Institute of Tropical Medicine, Antwerp, Belgium; 3 Médecins Sans Frontières Operational Centre Brussels, Luxembourg Operational Research Unit (LuxOR), Luxembourg, Luxembourg; 4 Division of Social and Behavioural Sciences, School of Public Health and Family Medicine, University of Cape Town, Cape Town, South Africa; 5 Department of Family Medicine and Population Health, University of Antwerp, Antwerp, Belgium; 6 Migration Health Specialist, Forced Migration Team, Analysis Department, Médecins Sans Frontières Operational Centre Belgium, Brussels, Belgium; 7 Present address: Institute for Global Health, University College London, London, United Kingdom; 8 Department of Natural Sciences, Lebanese American University, Beirut, Lebanon

**Keywords:** Refugees, Non‐communicable diseases, Diabetes, Health seeking behavior, Health service delivery, Health systems, Humanitarian response, Lebanon

## Abstract

**Background:**

Navigating health systems in host countries can be a challenge for refugees, particularly in a multi-provider system such as Lebanon. Syrian refugees in Lebanon face a high burden of Non-Communicable Diseases (NCDs) including diabetes mellitus. Evidence on how refugees navigate the health system is essential to improve provision of NCD services. We conducted a qualitative study amongst Syrian diabetes patients visiting Médecins Sans Frontières (MSF) clinics in one urban and one rural setting in Lebanon to explore factors influencing choice of and pathways to diabetes care.

**Methods:**

In-depth interviews were conducted with male and female adult participants with DM type 1 or type 2 who were receiving treatment at MSF clinics. Participants were recruited using convenience sampling. Interviews were conducted in Arabic and directly transcribed and translated into English. Data were coded in NVivo and analyzed using an inductive thematic approach.

**Results:**

A total of 29 in-depth interviews were conducted with 13 men and 16 women. Knowledge and understanding of diabetes management differed among participants. Syrian refugees in Lebanon gathered information about health services for diabetes largely from social networks of family and peers rather than through formal means. Pathways to care included different combinations of providers such as clinics, pharmacists and informal providers.

**Conclusions:**

Syrian refugees with diabetes in Lebanon face considerable challenges in navigating the health care system due to their vulnerable status and limited knowledge of the host country systems. To ensure access to care for diabetes, efforts need to be made to support patients’ orientation in the Lebanese health system.

## Background

Conflicts and crises increasingly affect middle-income countries (MICs) that have already experienced an epidemiological shift from infectious disease to Non-Communicable Diseases (NCDs) [1]. As physical injuries, forced displacement, degradation of living conditions and interruption of care caused by humanitarian crisis situations can lead to the severe deterioration of chronic disease, NCD care should be included in both emergency and protracted crisis contexts [2–4]. Health actors are increasingly aware that health care for refugees needs to focus on integrating care and health support beyond acute emergencies, as well as on primary care [5]. Integration of care is particularly challenging when refugees live in non-camp settings and access care through multiple providers, as is the case in Lebanon.

In 2018, Lebanon hosted approximately 1.5 million Syrian refugees; one in four of its residents is a refugee [6] and most live in non-camp settings. Approximately 53 % of Syrian refugee households in Lebanon report having at least one member with a chronic illness [7]. NCDs, including diabetes, are highly prevalent among the Syrian refugee population [8, 9]. Diabetes is a chronic disease that needs continuous, lifelong treatment, similar to other NCDs such as hypertension, or communicable diseases like HIV. If not followed up properly, kidney, eye, heart and nerve complications can lead to premature morbidity [10]. In humanitarian settings, additional morbidity and disability of diabetes could be avoided through adequate care provision [11].

The Lebanese health system is highly fragmented, with multiple providers including the Ministry of Public Health, Ministry of Social Affairs, political parties, non-governmental organizations (NGOs) and faith-based organizations. It remains largely dominated by the private sector, which offers 85 % of hospital beds in the country, and most specialized care [12].

Health access for Syrian refugees in Lebanon went through several organizational and administrative changes since the onset of the Syrian Civil War in 2011. Before 2015, the UNHCR and NGOs acted separately from the national health system. Thereafter, with funding support of UNHCR, health care for Syrian refugees was integrated into the Lebanese national assistance system, engaging the Ministry of Public Health and providing Primary Health Care (PHC), including NCD screening and care, through an existing network of Primary Health Care Centers (PHCC), most of them run by national NGOs [13, 14]. The PHCCs charge a fee of approximately 10 USD per visit and distribute some essential drugs and vaccines free of charge for the patient. Not all types of diabetes medication are available [13]. Regarding secondary and tertiary care, UNHCR partially covers the cost of life-saving treatments in hospitals, but the remainder needs to be paid directly by the patient [12]. Outside the official health system, Syrian doctors and health care workers often provide less expensive services [15] and some pharmacies sell medicine for diabetes or antibiotics without prescription [16]. However, accessing diabetes care for most Syrian refugees requires out of pocket payments, particularly for hospitalization, medication and diagnostics [7, 12, 13].

Faith-based organizations and national and international NGOs including Médecins Sans Frontières (MSF) strive to fill this gap and operate or support centers offering PHC free of charge or at low cost [17–19].

In this complex landscape of health care providers, Syrian refugees face challenges in accessing PHC, including care for NCDs [20–23]. While there are existing studies on patterns of health seeking behavior amongst Syrian refugees with chronic diseases in Lebanon, much of this research is quantitative or focused on health expenditure [7, 20, 24–27]. Less is known about the personal experiences of refugees in accessing NCD care and their perspectives on choosing providers.

MSF operates clinics in different regions of Lebanon, where it provides NCD care for vulnerable residents [17, 28]. To inform improvements to the implementation of the NCD program, we conducted a qualitative study among MSF patients. We explored how Syrian refugees seek health care for diabetes in the multi-provider setting of Lebanon by examining determinants and pathways of health seeking behavior.

## Methods

### Study design and sampling

This was a qualitative study, which was conducted in two sites in Lebanon in February and March 2019. Study participants included male and female Syrian refugees living with Diabetes Mellitus Type 1 (T1DM) or Diabetes Mellitus Type 2 (T2DM) who were receiving care from MSF clinics in Wadi Khaled or Shatila. We included adult refugees aged 18 years or above who resided in Wadi Khaled or the greater Beirut area and consented to be audio-recorded during the interview. Convenience sampling was used to recruit participants from the two clinics. We aimed at including a balance of male/female patients and those with different types of diabetes from both sites, as well as a varied age range. Patients present at the clinics during the study period were approached by a translator in the waiting room, informed about the study and invited to participate in an interview.

### Study setting and sites

MSF offers basic NCD care free of charge at its clinics in rural Wadi Khaled, Akkar and in urban Shatila, Beirut. These services include diagnostic tests, clinical care, provision of medication, referral costs for emergency care, patient support, education and counseling services and social services [17]. In Wadi Khaled, there are nine PHCCs, four of which offer NCD services, as well as several private doctors and pharmacies. In the more urban area of Greater Beirut, a range of health-care options exist.

Wadi Khaled is located in the north-east of Lebanon, on the border with Syria. Wadi Khaled has a registered Lebanese population of around 40,000 inhabitants, and approximately 25,000 registered refugees [29]. Wadi Khaled is among the poorest and most under-served regions of Lebanon. The Lebanese Armed Forces installed checkpoints upon entry to and exit from Wadi Khaled and refugees are often unable to cross. In June 2018, the active NCD cohort at the MSF clinic in Wadi Khaled included 31 patients with T1DM and 222 patients with T2DM.^3^

Shatila is originally a Palestinian refugee camp located in the capital city, Beirut. According to UNHCR estimates, Beirut and the surrounding Mount Lebanon area now host around 240,000 registered and 90,000 unregistered Syrian refugees, with the highest clusters in South Beirut [30]. Most Syrian NCD patients visiting the MSF clinic come from outside Shatila, largely from the Greater Beirut area. In December 2017, the active NCD cohort of Syrian patients at the MSF clinic in Shatila included 204 patients with T1DM and 1650 patients with T2DM.

### Data collection

Interviews were conducted at the MSF clinic in each site or at the home of the interviewee, depending on the preference of the individual. The mean duration of interviews was 50 min. In-depth interviews (IDIs) were conducted by the female principal investigator (PI) (FH) using a piloted semi-structured interview guide with open-ended questions that explored the healthcare seeking experience of participants since their arrival in Lebanon, including (1) information sources for health and health care providers, (2) health care seeking pathways, and (3) factors influencing choice of pathway (Appendix 1). Interviews were stopped when saturation was reached. The PI was supported by trained translators (one male and one female) who were fluent in Arabic and English. Neither the translator nor the PI were involved in direct patient care. Patients were reassured that their participation was voluntary, and that it was not linked to the care they were receiving from MSF. Several patients who were approached in the waiting room refused participation. If patients agreed, they were invited to the interview room where potential harms, voluntariness and confidentiality were discussed during the informed consent process. Two potential participants refused participation after the informed consent process.

### Data analysis

All audio-recorded IDIs were transcribed and translated from Arabic to English by an external transcriber, reviewed by the PI then discussed with the translators and transcriber for verification. A coding framework was developed in NVivo (QSR version 12), based upon content analysis of the data. Each code referred to an underlying theme, and themes were grouped into larger concepts that emerged from the data analysis. The PI coded the transcripts which were then reviewed by another co-investigator (EV) and discussed with the rest of the study team. In order to answer questions of factors influencing the choice of provider and health care seeking pathways among Syrian refugees in Lebanon with diabetes, we chose content analysis to allow themes to emerge from the transcribed data. Following the inductive coding of answers and the identification of themes, the analysis was then informed by theoretical approaches to factors influencing health seeking behavior to further interpret and categorize the answers.

## Results

In total, 29 patients participated in the study and of those, 13 were female and 16 were male (Appendix 2). Participants ranged in age from 19 to 80 years, with a median age of 50 years. The median age of the 11 participants with T1DM was 29 years, and for the 18 participants with T2DM, 57 years.

The main themes emerging from the data were:

1. Awareness and understanding of diabetes treatment and services;
2. Factors influencing choice of provider; and
3. Different health care seeking pathways.

### 1. Awareness and understanding of diabetes treatment and services

#### Subjective knowledge of diabetes disease and care

Participants described how their own and their family’s knowledge about diabetes influenced which type of care provider they chose, and how they follow up and manage their disease. In general, participants had a good understanding of the bio-medical concepts of diabetes and the lifestyle factors influencing its progression, though some described a lack of knowledge, and how this then influenced the way they sought follow-up and care. Those unaware of the importance of regular monitoring reported delaying seeking care until experiencing acute symptoms or a worsening of their condition.

“[My diabetes] wasn’t stable when I was young. I told you, I didn’t know about eating rice and white bread. All of this increased the diabetes levels. I didn’t measure my glucose levels all the time. In Syria, I used to go to the PHC once every two to three years. Now, here with MSF, I am following up more. [In Syria], maybe it was a little bit of neglect from my parents and I was at school, I don’t know. I did not know a lot about the disease. I did not know what it was, what it meant, and its complications. I used to know that I had to take the medication, eat normally and cut out sweets only. I did not know that carbohydrates increased the glucose level. I did not know any of this information. (…) We did not follow up on the disease and we did not know that it had these complications. When we began to go and follow up with the doctors [at MSF], I had a better knowledge about diabetes.” (woman with T1DM, 24 years old, Wadi Khaled).

#### Awareness of available services for diabetes care

Participants in both Wadi Khaled and Shatila had differing degrees of knowledge of care providers. They described how their choice of care-provider was influenced by knowledge gained through their family and peer networks. Most interviewees learned about the existence of different health care providers from family members, Lebanese and Syrian neighbors and friends. They described how people who were already seeking care from a particular provider shared this information and made recommendations to them.

“I was at my friend’s house and there was a guy who came from Syria who has diabetes. So, my friend said that he had diabetes and it is improving; he also told us that he is going to MSF and they are giving him medication and he got better.” (man with T2DM, 42 years old, Wadi Khaled).

“When someone is sick, they would always ask people around them.” (woman with T2DM, 60 years old, Wadi Khaled).

#### Awareness of available services for diabetes-related care

Interviewees used the same channels of information that they used when seeking general diabetes care to find a specialist such as an ophthalmologist for diabetes-related complications or a gynecologist specialized in the pregnancy of women with diabetes. Three participants mentioned learning about providers through adverts on social media or posters, and home visits by MSF community outreach staff, while several found out about a new provider “by chance.”

“Concerning diabetes, I’m here [at the MSF clinic] and I would never change, but concerning other things, such as eye tests, we usually go to [a PHCC] on the old airport road, it costs very little. (...) [I chose it] because I don’t know anywhere else. I also know a girl who works there, she is Syrian and she told me it is very good.” (man with T1DM, 45 years old, Shatila)

### 2. Factors influencing choice of provider

Several factors influenced the patient’s choice of health care provider for diabetes: quality, distance, economic factors and their legal status.

#### Perceived quality

Perceptions of the quality of care were important considerations when choosing a health-care provider. Perceptions relating to quality were shaped through people’s own experiences as well as recommendations of specific doctors, tests, facilities and medication made by members of their social networks.

“I care about the doctor being good medically, as well as people talking about his achievements… I care about the doctor’s experience and his good reputation among people.” (man with T2DM, 65 years old, Shatila).

“There was a woman here that had diabetes, and she told us that [the doctor’s] service is really good and that he takes good care of the patients… and he does a full checkup. To choose a doctor, I ask around. They might tell you that X and Y went and it was good. If it was bad, I don’t try it. I try the one that they say is good. (…) I did not really like [this PHCC]. Their treatment is not humanitarian. They don’t treat you nicely. You feel that they barely give you the service, from the way of talking and treatment. I am talking about the employees, not the doctors. (…) When [another PHCC] opened, I stopped going there.” (woman with T2DM, 60 years old, Wadi Khaled).

Relevant considerations for assessing quality were the improvement of health status (both subjective, as “feeling better”, and objective, such as a decreased HbA1C level), communication and attitude of health care staff and the uninterrupted availability of medications.

“It is not about choosing an option. I go try the doctor, if I see that I am getting results, I would carry on going to him.” (woman with T1DM, 27 years old, Shatila).

“The only doctor that told me that diabetes doesn’t go away is the one from [a city] in Syria. (…) His treatment was really good, but the only negative thing was the insulin in Syria. The electricity was really weak and the insulin was not in the fridge. That’s the only reason I came here [to the MSF clinic], it’s better.” (man with T1DM, 19 years old, Shatila,)

“We used to go to [a PHCC], and every once in a while they gave us the medication. They stopped giving us the medication, so I stopped going. I want to be honest. I went the first time and they gave me the medication, the next time they gave me the medication was four months later.” (woman with T2DM, 80 years old, Wadi Khaled).

Providers from the private sector were perceived to have the highest quality of care, especially those that were expensive, as well as MSF clinics.

“He told me that [MSF] provided really good care and an amazing service. They treat you for whatever you have and they treat you like a private doctor.” (men with T2DM, 65 years old, Shatila).

“If you had a lot of money, you would have the best treatment. Everyone wishes that.” (woman with T1DM, 30 years old, Shatila).

#### Economic factors

Both direct health costs (consultation fees, purchase of medication, diagnostic tests etc.) and indirect health costs (transportation, work loss, etc.) were considered essential when deciding where to seek services for diabetes. In Wadi Khaled, several participants described having difficulties paying for transportation to the health facility, or finding affordable transportation. If services were “free of charge”, this was an important determinant for choice, especially if people had co-morbidities. Several patients valued regular follow-ups with clinicians and monitoring tests, but when out of pocket payments were required, they would buy medication directly from pharmacies, change provider or miss routine visits instead. In some cases, economic challenges and lack of knowledge of the availability of free care services led patients to have periods without medication. Some interviewees mentioned financial factors as a reason for deferring care for diabetes-related complications.

“I stopped taking medication for two years (…) here in Lebanon, because it wasn’t available for free. (…) I couldn’t afford it. When we first came here, we used to buy it. We had money. Later, I went to [a hospital] and got pills for two months, and when [MSF] opened here, I came here. I am taking my medication from here, monthly.” (man with T2DM, 76 years old, Wadi Khaled).

“Sometimes I couldn’t buy it. I stopped taking it until I could buy it again, we have other priorities at home which are more important. So sometimes I would stop taking the medication for ten days until I could afford it again. (…) My husband is a worker and sometimes he did not have the money for the medication, and we had children, so we had daily needs for the house which were more important.” (woman with T2DM, 45 years old, Shatila).

“Back then, I was working, I used to make money and buy the medication. My children used to help me and there were some people that helped me by providing me with the medication… I told the doctor that I need the cheapest medication. The medication was for 5,000 Lebanese Lira (3,3 USD). My wife’s brother buys the diabetes medication that costs 80,000 Lebanese Lira (53 USD). I prefer buying the cheapest one.” (man with T2DM, 65 years old, Shatila).

#### Distance and transportation

Interviewees from urban Shatila did not mention distance or transportation as a barrier to accessing care, but several participants in rural Wadi Khaled described difficulties finding transportation, including in emergency situations or times of acute illness. If someone wasseverely ill and was unable to travel, they would consider changing provider and going to their nearest PHCC or buying medication directly from a pharmacy.

In Wadi Khaled, several female interviewees highlighted the importance of proximity when choosing a PHCC, with many of them dependent on a male family member to accompany them to clinic visits. If the distance to the facility was large and took more time, male family members were less available to accompany their relatives as they could not afford to take time off work.

“I only went once; I did not feel comfortable… The distance is far. (…) And the transportation is hard. Whenever I think about it, I just stay at home and I do not go.” (woman with T1DM, 27 years old, Wadi Khaled).

“I don’t go if [my brother and father] don’t take me, (…) sometimes it is hard. If my brothers are at work, they need to take the day off to take me. Sometimes they cannot, because they need to work to make a living. We only go where it is near, because diabetes needs follow up so I always look for something close.” (woman with T1DM, 24 years old, Wadi Khaled).

#### Legal factors

The legal status of the Syrian interviewees also had an influence on where they chose to access health-care services. Some participants considered seeking care in Syria for diabetes-related complications but their legal status within Lebanon and official and unofficial border fees made this challenging.

“I had a sponsor, so I used to go [to Syria] in an official way, but now I haven't had a sponsor for the last three months, so my papers are not official… I couldn’t go [to Syria] for checkups every two months, it is cheaper, but I do not live there and I could not leave [Lebanon].” (woman with T2DM, 45years old, Shatila).

“No, I never went back to visit my doctor in Syria. I came here [to Lebanon] legally but then I broke it. If I went to Syria and wanted to come back to Lebanon I had to pay 900 USD. I should pay because I am here for a visit and I stayed more. (…) If I wanted to go back to the doctor, I would face obstacles.” (woman with T1DM, 27 years old, Wadi Khaled).

In Wadi Khaled, legal status was also relevant, as some male participants would not cross a military checkpoint due to security concerns, which limited their access to care.

“The issue is if you are not legal. I am not legal, but it doesn’t stop me. To some people, it is a problem if you are illegal. It’s rare that they [the military] take [detain] someone. If you are heading to the hospital, they won’t stop you.” (man with T2DM, 42 years old, Wadi Khaled).

### 3. Health care seeking pathways

Interviewees described seeking care from different ‘combinations’ of health-care providers, and rarely received all elements of required care from one provider.

#### Seeking care on arrival

Upon arrival in Lebanon, many participants described a period in which they *“didn’t know anything”* about the health system and humanitarian assistance support available to them. This led to them buying their medication at a pharmacy in Lebanon or arranging its purchase in Syria and transportation to Lebanon. Patients would then ask their social networks of family members and peers for advice if they were no longer able to access or afford medication through this initial route. In other cases, interviewees learned about the availability of other providers through their social networks “by chance” and subsequently changed to a different facility:

“I had the card that I used in Syria that had the insulin type and dosage on it. When I came [to Lebanon], I went to a pharmacy, I did not go to a diabetes doctor. I showed the pharmacist the card. I stayed like that for a while. We have been in Lebanon for 8 years, I stayed like that for almost 3–4 years. Then there were people that came to our building, and they told my daughter-in-law about MSF.” (woman with T1DM, 54 years old, Shatila).

#### Seeking care from multiple providers

Interviewees often sought care for diabetes from more than one provider at the same time, or changed providers based on challenges accessing their first choice of provider, unstable provision of medication or inability to pay. Consultations and prescriptions were often given at PHCC level, and medication then collected from a different pharmacy. In some cases, interviewees accessed a pharmacy as their principal provider and only went to a clinician in the case of acute symptoms.

“I haven’t visited [the doctor] in a long time. I stopped when my results became stable.” (woman with T2DM, 47 years old, Shatila).

“I didn’t see a doctor, I used to take my medication, and sometimes I used to go to the pharmacy to test my glucose. I only did the test for Hb1AC twice at the private doctor and that was two years ago… But in the last period, I felt tired and I thought that I had to do tests and see a doctor.” (woman with T1DM, 54 years old, Shatila).

Sometimes, interviewees received a basic package of diabetes care (including medication, testing and consultation) free of charge or at a subsidized price at a PHCC, then visited a private specialist if they were pregnant or saw a change in their condition. Some described how they would attempt to obtain medications from other providers if drugs were not available at their usual PHCC. They also reported accessing services outside of their regular provider in the case of illness, when they would go to the closest facility. Seeing an improvement in their health was also reported as a factor for staying with a specific care provider for regular diabetes care, medication or care for diabetes complications.

Three case stories of participants illustrate examples of health care seeking pathways.

#### Case story of a 60 year old man with T2DM, living in Wadi Khaled

AB is 60 years old. He came to Wadi Khaled from Syria in 2012. He has hypertension and T2DM. “When I came here, I had around three boxes of medication with me from Syria (…). They lasted me for five to six months. After that, the Lebanese people told us about the PHCC in the area to treat diabetes.“ He first went to a PHCC in F., quite far from his house, that provided care and medication free of charge. But after some years this facility closed. He changed to PHCC N, where consultation cost was low, but AB struggled to pay for tests and medication. When he learned that the MSF clinic provides comprehensive care free of charge, he accessed MSF’s services instead. “I don’t have money, if I go to a doctor or to the PHCC N, they would give me a prescription to go buy my own medication from the pharmacy. They don’t give you medication for free. Since we came here, our financial situation is not really good. For the lab test, they wanted 40 USD; where do I get that from? (…) If you don’t have the money, they would not look at you.“ AB experienced an episode of acute illness that hindered his access to care at the MSF clinic: “I kept on going to MSF to get treated until one day I got sick. I couldn’t go to MSF anymore. I gave my brother the card, MSF did not give him [the medication for me]. They told them that they wouldn’t give it to him unless I was there. That is their right. I stopped going for four to five months and [my siblings] got me the medication from here and there. And people helped us. (…) My siblings got me a box or two from a pharmacist. Sometimes [the pharmacist] did not take money, sometimes he did. I stayed like that for four months, and my diabetes was getting worse. I couldn’t take it anymore; I came back to MSF.“ Now, AB combines the services of two providers. Once per month, he attends his regular appointment at the MSF clinic for consultations with the doctor, tests and the collection of his medication. If he feels acutely unwell and wants to check his blood pressure and glucose level, he goes to yet another PHCC near his house, where those tests are free of charge. His doctor at MSF told him about this option. If the test results are abnormal, he seeks immediate treatment at MSF. AB chose this combination because he struggles to pay for transportation to reach the MSF clinic.

#### Case story of a 22‐year‐old woman with T1DM, living in Wadi Khaled

Some years ago, PS felt unwell with diabetes symptoms. She visited three health care providers over the course of several months, but they all failed to diagnose her correctly. One provider asked her to pay for diagnostic tests, but her family could not afford them, so she did not return for follow up. Several months later she collapsed and was brought to the hospital outside Wadi Khaled for emergency care. T1DM was diagnosed and slow and rapid insulin was prescribed. PS then bought slow and rapid insulin at a pharmacy and sometimes went to PHCC X in Wadi Khaled where she received mixed insulin free of charge. The PHCC only provided mixed insulin but PS needed slow and rapid insulin, and the provision was not stable. At this PHCC, PS met Dr. Z who recommended that she go to the nearest MSF clinic (at this time, 60 km outside Wadi Khaled) because MSF would provide her with best available care at no charge. PS trusted Dr. Z and consulted her about any decisions that might impact the disease. After the long delay in diagnosis, the most important thing for PS at this point was to see a good doctor. She was willing to travel a long distance and pay for good care. She went to the MSF clinic near Tripoli twice, but stopped because she felt the distance was too far. She continued obtaining insulin from pharmacies and from the PHCC X, and followed up with Dr. Z (who left Wadi Khaled) over the phone for the period of one year.

As soon as MSF opened an NCD clinic in Wadi Khaled, PS registered. For some time, MSF was her only diabetes care provider. Later, Dr. Z opened a private practice in Wadi Khaled near the patient’s house. Now, PS feels comfortable with the care at MSF and she sees Dr. Z in case of acute illness or emergencies. Dr. Z offers her advice free of charge to PS.

#### Case story of a 47‐year‐old woman with T2DM, living in Greater Beirut

FM already lived in Lebanon when she first experienced symptoms. She went to a laboratory that is well known amongst her peers in the area she lived in, to get tested. After the positive test, she went to a PHCC and asked for referral to a specialist “doctor for diabetes”. She chose that PHCC because it was near to her house, and cheaper than other places, “they treat people in a good way”, and it has “all the doctors we need”. FM says: “They have different doctors, and if the doctor that does not come to the center, we can go visit him/her in his/her clinic after paying at the center.” FM visited the diabetes specialist at her private clinic. She had a positive experience: the doctor was “really great, she treats people well, she takes care of the patient.” Once FM’s blood glucose levels were stable with medication, she stopped seeing the doctor. In the next 1.5 years, FM followed the doctor’s prescription and bought medication from the pharmacy. Then she heard from peers that “at MSF there are doctors and they provide medication and they treat people really well”. As FM suffers “from a chronic disease and always need[s] medication”, she “can’t always afford it”. FM says: “When I first had diabetes only, it was okay. When I was diagnosed with hypertension, I had six different types of medication to take. So it was hard. And I have to constantly run tests.” Now her only care provider for diabetes and hypertension is MSF, but recently she got diagnosed with anemia. This anemia medication is not available at MSF, so she buys it at a pharmacy.

## Discussion

Our study adds a new perspective to the existing knowledge on health care access and the utilization of services within refugee populations, namely how people in a multi-provider health system choose to seek care and services for a chronic disease such as diabetes. The findings show that health seeking behaviour is influenced by an individual understanding of diabetes and its management, perceptions of quality of care and contextual constraints such as transport and legal barriers. Acute episodes and co-morbidity contribute to the variability in health seeking pathways.

The subjective level of knowledge about diabetes and its management varied amongst the Syrian refugees in our study, which partially confirms other studies showing that diabetes patients from the Middle Eastern region have generally limited knowledge about disease management [31, 32]. Other studies showed that Syrians who were diagnosed after the onset of the crisis have less knowledge than those diagnosed prior to displacement [33]. People with lower levels of knowledge about diabetes are known to be less adherent to treatment [34, 35] and adequate self-management [36]. Irregularity of follow up visits also pose an increased risk for diabetes complications [37].

This study shows that Syrian refugees lack orientation in health-care services in Lebanon when they arrive, and may still rely on their social networks to obtain their medication from Syria before accessing the Lebanese health system. We found that Syrian refugees in Lebanon gain information about health services for diabetes largely from family and peers rather than through formal means, such as health-care providers or the UNHCR. Similar behaviors have been documented in refugee populations in other settings [38]. In the case of Syrian refugees in Lebanon, reasons for this might include inadequate information channels, experiences of unclear information, mistrust in public institutions and perceived discrimination in the health system [21].

Whilst the urban camp of Shatila and the rural setting of Wadi Khaled have different provider landscapes, we did not find pronounced differences in approaches to seeking care based to location. Similarly, we did not find pronounced differences between the health-seeking behavior of individuals with T1DM and T2DM, even though the disease types differ in pathology, progression and treatment. It seems that the other factors presented above are more prominent in determining care pathways, but more research is needed to better understand this issue.

Among participants in this study, the parallel use of different combinations of health care providers was common as a result of limited knowledge, access barriers, and the structure of the health-care system. McNatt et al. found similar evidence in Jordan, where Syrian refugees usually visited more than three facilities for NCD care because of the narrow selection of services available at each institution [39]. In other settings, chronic disease and unsolved health problems [40] have been seen as positively associated with visiting multiple doctors for the same health problem, while trust in the current provider [41] and satisfaction with treatment contributes to not seeking care with other providers [42]. Receiving care from more than one provider in parallel can cause detrimental health outcomes, and attending more than one clinic for follow up has been found to be a risk factor for diabetes complications among Egyptian patients [37] and in other populations [43].

Socio-economic status and legal status posed structural barriers to accessing health care for many of our participants. Amongst the identified factors influencing choice of provider, we found that financial considerations were a main factor for those with co-morbidities and chronic conditions, who favoured providers that offered medication and treatment free of charge. Coping strategies for financial barriers, such as delaying doctor’s visits and buying medication directly from pharmacies have also been documented among Syrian refugees in Jordan [39] and cost has been previously identified as the main barrier for accessing health care for chronic conditions for Syrian refugees in Lebanon [18, 20, 26]. Despite the efforts of many actors to enhance access to care, Syrian refugee’s find it increasingly difficult to afford care (for any condition) [26], possibly because share of out of pocket payments is increasing, and economic conditions are further deteriorating. In 2019, about half of Syrian refugee households were living in extreme poverty [23]. In the light of high financial vulnerability and risk of catastrophic health expenditure, access to comprehensive care should be ensured with limited co-payments, especially for those with multi-morbidities and chronic conditions that require long-term treatment.

We found that Syrian refugees face difficulties navigating the Lebanese health system and accessing adequate care. On one hand, this is due to system level factors: the health system is fragmented and complex, and comprehensive information about the availability of and access to health services is difficult to provide [21]. Syrians refugees in Lebanon face an additional challenge as result of transitioning from a one-provider national health system in pre-war Syria [44] to a fragmented multi-provider system. To facilitate the orientation of refugees in a new health system, information about health services needs to be easily accessible and understandable. The concept of “health literate health care organizations” can serve as suitable approach for health care organizations to address this issue on a system level [45]. On the other hand, the difficulties in navigating the health system are caused by individual factors, such as insufficient level of patient awareness and understanding of diabetes treatment and services in this specific context. A lack of health literacy amongst patients has been previously shown to contribute to the challenges of navigating the health system of a host country [38, 46]. As suggested by others, [12, 18] we find that Syrian refugees need better support to identify and access adequate NCD care. Health care providers should engage in raising awareness about existing services and directly assist Syrian refugees to identify and access care, as well as in improving the diabetes literacy of patients, for example through providing diabetes self-management, education and support programmes [33, 47, 48].

It is important for health-care providers to recognize that seeking treatment from multiple providers is common among this population. Including discussions about care received from other providers should be part of routine patient consultations, to reduce the high risk of drug interactions, to limit potentially harmful self-management strategies like adjustment of dosage, and to foster continuity of care among a patient group with low diabetes literacy. Further research is needed to fully understand the reasons for and implications of combining providers for diabetes care amongst Syrian refugees in Lebanon, as well as for refugee populations in other settings. In situations such as that of Syrian refugees in Lebanon, where a vulnerable population struggles to navigate a complex multi-provider system, health care providers need to further coordinate the services offered and organize care in a way that is easy for the patient to understand and access, whilst ensuring continuity of care.

The following study limitations need to be taken into consideration. Firstly, we only recruited patients from MSF facilities, thus their views may not represent those of community members who do not access MSF’s services. While results of this descriptive study are specific to the population and settings it was conducted in, findings may also shed light on related practices and experiences, for example for Syrian refugees accessing care for other chronic conditions in Lebanon, or vulnerable refugees accessing diabetes care in similarly fragmented health systems.

Secondly, the PI and the translators who conducted interviews were affiliated with MSF, even though they were not directly providing care to patients. This means participants may not have felt comfortable to criticize MSF, thus bringing in a degree of social desirability bias. We strove to mitigate this risk by emphasizing the confidentiality of anything said within the interview during the informed consent process.

Finally, the PI does not speak Arabic and was reliant upon translators. This limitation was mitigated through thoroughly reviewing the interviews and the transcripts to ensure that the loss of nuances during translation was minimal.

## Conclusions

Syrian refugees with diabetes in Lebanon face considerable challenges in navigating the health care system which leads to reduced access and interrupted disease management. Continuity of care for this vulnerable population, and for people with other chronic diseases, needs special attention. Initial steps could be to support patients in Lebanon in their orientation within a multi-provider health system and encourage providers to ask patients about their pathways to care in order to help them access treatment and follow-up visits.

## Data Availability

Not applicable.

## Appendix 1

**In-depth interview guide**

- Introduction

Introduce yourself and the translator.

Thank you for agreeing to talk with me today. I would like to ask you some questions about your experiences regarding health care for diabetes. The interview will take approximately 30 min to 1 hour. There are no right answers or wrong answers to the questions I will ask you. If you don’t feel comfortable answering some questions you have the right not to answer. We can also stop this interview at any time.

- Can you tell me about yourself?

P: Gender, Age, Residence (village), Nationality, Refugee status, Disease

- When and where did you first find out about your diabetes, and what happened next?

P: diagnosis in Lebanon or Syria, accidental diagnosis, type of diabetes, caregiver. Treatment: insulin-dependent or not

- How did/do you find out about where you could go for health care for diabetes? (Where can you get treated?)

P: sources of information: family, friends, neighbor, community leaders. Knowledge of services, availability of services, accessible services (restricting factors)

- Now, where do you go for health services for the diabetes?

P: different health seeking behavior in different situations. Where do you go when you feel you have a problem with the diabetes? Where do you go if you feel you need to see a doctor? Where do you go to get your drugs? Where do you go for emergencies relating to diabetes?

- Before, did you go somewhere else for diabetes care?

P: pathways of accessing care. Factors influencing “ending up” at a certain provider.

- Was there a time when you couldn’t come to your usual/preferred provider/the MSF clinic? Why was that so? Can you tell me where you went instead?

P: pathways of accessing care. Alternative care providers. Restrictions in accessing care.

- What do you like about this doctor/PHC/hospital? What did you not like about this doctor/PHC/hospital? Why did you choose this doctor/PHC/hospital?

P: Explore factors influencing choice. (see below)

- Generally, what is important for you when you go to a doctor/ to a clinic?

P: Explore factors influencing choice: cost, location, reputation, opening times, waiting times, gender of staff, international staff vs. local staff, confidentiality, trust, quality, communication skills of staff, (branded/generic) drugs, availability of drugs

- Do you go to different doctors/clinics for different services for diabetes? (and why?)

P: Multiple providers, different availabilities.

- What is the preferred place you would go?

P: factors of choice, preferences.

- If you would have 2 options, which factor would be more important to you?

P: factors of choice, preferences.

- When you have a question regarding diabetes, who do you ask? And why?

P: sources of information, decision-takers in the community, trust in health care providers

## Appendix 2

**Table 1:** Case characteristics and overview

**Shatila**				**Wadi Khaled**			
*case*	*gender*	*DM*	*age*	*case*	*gender*	*DM*	*age*
1	female	1	27	17	female	1	22
2	female	1	30	18	female	1	24
3	female	1	54	19	female	1	27
4	female	2	39	20	female	2	60
5	female	2	45	21	female	2	70
6	female	2	47	22	female	2	80
7	female	2	49	23	male	1	50
8	male	1	19	24	male	2	42
9	male	1	29	25	male	2	50
10	male	1	33	26	male	2	58
11	male	1	45	27	male	2	59
12	male	2	53	28	male	2	60
13	male	2	55	29	male	2	76
14	male	2	55				
15	male	2	59				
16	male	2	65				
*Case characteristics*							
	*Type 1*		*Type 2*				
**Shatila**	4	3	5	4	**16**		
**Wadi Khaled**	1	3	6	3	**13**		
	*11*		*18*				
*Case overview*							

## Abbreviations

DM: Diabetes mellitus
IDI: In-depth interview
MIC: Middle-income countries
MSF: Médecins Sans Frontières
NCD: Non-communicable disease
NGO: Non-governmental organization
PHC: Primary health care
PHCC: Primary health care center
PI: Principal investigator
T1DM: Type 1 diabetes mellitus
T2DM: Type 2 diabetes mellitus
UNHCR: United Nations High Commissioner for Refugees

## Footnotes

3 In Wadi Khaled, numbers for Syrian patients are not available due to database limitations. We estimate that about half of the patients are of Syrian nationality.
